# Price versus clinical guidelines in primary care statin prescribing: a retrospective cohort study and cost simulation model

**DOI:** 10.1177/01410768211051713

**Published:** 2021-11-18

**Authors:** Matias Ortiz De Zarate, Emmanouil Mentzakis, Simon DS Fraser, Paul Roderick, Paul Rutter, Carmine Ornaghi

**Affiliations:** 1 Department of Economics, Faculty of Social Sciences, University of Southampton,Southampton SO17 1BJ, UK; 2 School of Primary Care, Population Sciences and Medical Education, Faculty of Medicine, University of Southampton, Southampton General Hospital, Southampton SO16 6YD, UK; 3 School of Pharmacy and Biomedical Sciences, Faculty of Health and Science, 6697 University of Portsmouth, Portsmouth PO1 2UP, UK

**Keywords:** statins, prescribing behaviour, clinical guidelines, patent expiration

## Abstract

**Objective:**

To investigate the relative impact of generic entry and National Institute for Health and Care Excellence clinical guidelines on prescribing using statins as an exemplar.

**Design:**

Retrospective analysis of statin prescribing in primary care and cost simulation model.

**Setting:**

Royal College of General Practitioners Research and Surveillance Centre (RCGP R&SC) database and Prescription Cost Analysis (PCA) database.

**Participants:**

New patients prescribed statins for the first time between July 2003 and September 2018.

**Main outcome measures:**

Shares of new patients prescribed one of the five statins available in the British National Formulary, and cost of prescribing statins to new and existing patients in primary care in England.

**Results:**

General trends of statin’ prescriptions were largely driven by a decrease in acquisition costs triggered by patent expiration, preceding NICE guidelines which themselves did not seem to affect prescription trends. Significant heterogeneity is observed in the prescription of the most cost-effective statin acrossGPs. A cost simulation shows that, between 2004 and 2018, the NHS could have saved £2.8bn (around 40% of the £6.3bn spent on statins during this time) if all GP practices had prescribed only the most cost-effective treatment.

**Conclusions:**

There is potential for large savings for the NHS if new and, whenever possible, ongoing patients are promptly switched to the first medicine that becomes available as generic within a therapeutic class as long as it has similar efficacy to still-patented medicines.

## Introduction

In a context where national health systems of all high- and medium-income countries are confronted by ballooning costs of caring for an ageing population and an increase in prevalence of long-term conditions, promoting cost-effective prescribing represents an important part of controlling healthcare expenditure.^1,2^ In the English National Health Service (NHS), the National Institute for Health and Care Excellence (NICE) publishes national guidance aimed at promoting clinical and cost-effective evidence-based recommendations for the clinical management of different conditions. In therapeutic markets where treatments have similar safety and effectiveness, NICE recommendations may vary over time following changes in acquisition costs, e.g. due to patent expirations and the ensuing entry of generics. However, persistence of prescribing habits and prescribers’ lack of awareness of medicines’ actual cost may mean that the uptake of NICE recommended medicines can vary substantially across general practitioners and practices, despite efforts at local level, including Clinical Commissioning Groups (CCG), to encourage more cost-effective prescribing.^3–7^ Since low responsiveness to adopt NICE recommendations can substantially undermine NHS efforts to contain drug expenditure, it is important to understand the pervasiveness of such behaviour.

Statins represent an ideal market to investigate the relative importance of prices and clinical guidelines in explaining prescribing behaviour for at least two reasons. First, statins are the most widely used treatment for primary and secondary prevention of cardiovascular disease, conditions with an estimated cost to the NHS of roughly £7.4 billion a year.^8,9^ Second, there are five main events that have shaped the statins market over the last two decades. In May 2003, simvastatin (brand name *Zocor*) lost patent protection and became available as a generic drug. In January 2006, NICE published Technological Appraisal 94 (TA-94), stating that all statins were equally effective from a therapeutic point of view, advising general practitioners to consider costs of statins when choosing the initial treatment and advising that simvastatin was the cheapest.^10^ Clinical Guideline 67 (CG-67), released in May 2008, stated that treatment initiation should start with simvastatin.^11^ In May 2012, atorvastatin (brand name *Lipitor*) lost patent protection and became available generically. Finally, two years later, in May 2014, NICE published Clinical Guidelines 181 (CG-181) recommending atorvastatin as initial treatment.^12^ The reduced cost after patent expiration coupled with its relatively greater potency made atorvastatin the most cost-effective statin in the market.

Using statins as an exemplar, this study investigated the prescription dynamics in a large sample of the English primary care sector between 2004 and 2018. First, we explored the relationship between aggregate prescription trends and two sets of events that shaped the statin market: patent expirations and generic entry on the one hand, and publication of national clinical guidelines on the other. Second, we investigated variation in prescribing activity across general practices. Third, we quantified the forsaken savings for the NHS by assuming perfect therapeutic substitution, that is by comparing actual treatment choices to a hypothetical scenario where only the most cost-effective treatments are prescribed.

## Methods

### Data

Our data are retrieved from Royal College of General Practitioners Research and Surveillance Centre (RCGP R&SC) database, a nationally representative sample of 243 general practices in England. The population representativeness of this database has been addressed in previous studies, including its representativeness of the distribution of cardiovascular disease in England.^13,14^ From this database, we retrieved all first prescription episodes for more than 400,000 patients treated with statins between Q3-2003 and Q4-2018. This database contains complete information of each prescription issued and an anonymised identity code of the general practice issuing the prescription.

We also retrieved from the *Prescription Cost Analysis* (PCA) database, yearly statistics on the total *quantities* of each drug prescribed in primary care in England, with the corresponding total *spending*, obtained from net ingredient cost (NIC).^15^ This is the basic cost of a drug as used in primary care. NIC is used in Prescription Services reports and other analyses, as it standardises cost throughout prescribing nationally, and allows comparisons of data from different sources.

By aggregating the total spending of each strength of statin prescribed and dividing it by the corresponding total quantity, we obtained a measure of the average acquisition cost per strength of each statin in each year. In Appendix A, we compare the prescription data in the PCA dataset to those in the RCGP R&SC dataset to demonstrate that the latter constitutes a representative sample of national prescription of statins.

### Trends and heterogeneity in statins prescription

Since 2003, the statins market has experienced five exogenous changes to prices and clinical standards as explained above that may have triggered changes in general practitioners’ prescribing choice. To document how prescription trends change in proximity of those events, we plotted the average proportion (across the 243 practices in the RCGP R&SC dataset) of new patients starting with one of the five statins for the period 2004 to 2018, as well as the average acquisition cost per defined daily dose for each statin. To explore heterogeneity in prescription patterns, we split general practices in the RCGP R&SC dataset into quintiles for every month in the data according to their share of new patients treated with simvastatin and plotted the average shares of new patients treated with simvastatin in each of the resulting five groups. Although this offers an insight into the evolution of overall heterogeneity in the data, it does not allow us to characterise persistency in general practitioners’ prescribing choices. Hence, we additionally plotted the average share of new patients treated with simvastatin, keeping the composition of groups fixed at the quintile computed at Q3-2003.

### Cost savings simulation

According to NICE, all statins are equally therapeutically effective. As stated in TA-94 (2006), ‘from the evidence available […] [and] for the purpose of initiating therapy, there were no data on clinical events to suggest the superiority of any one Statin over all the others in reducing cardiovascular events’.^10,16-20^ Under the assumption that general practitioners cannot consistently anticipate whether a new patient would benefit from starting treatment with any given statin different from the one recommended by NICE, we evaluated prescription decisions according to a cost-minimisation criterion. Specifically, we quantified the potential savings for the NHS by comparing the actual cost of the observed prescription decisions with a hypothetical cost constructed by substituting the actual original treatments with a therapeutically similar treatment containing either simvastatin (before May 2012) or atorvastatin (from May 2012 onwards). By computing the difference between actual and hypothetic costs, we obtained a measure of the potential savings, both in absolute and relative terms.

The cost simulation was performed under two different scenarios. In the *first scenario*, the analysis was limited to the first 28 days of treatment for patients newly treated with statins. By focusing only on the first prescription episode, we compared the evolution of the spending on statin treatments using the same unit on analysis in different time periods, leaving aside the problem of following patients throughout their drug-treatment history. Clearly, the absolute value of savings obtained by considering only the first prescription episode for new patients is a partial account of the overall potential savings, as patients treated for cardiovascular disease risk will usually be on treatment indefinitely.

For this reason, we considered a *second scenario* where we computed hypothetical savings if practitioners had changed *all* (new and ongoing) patients to simvastatin (up to May 2012) and atorvastatin (after May 2012). This second simulation can be considered an upper bound to the absolute savings under the strong assumption that existing patients could be immediately switched to the NICE-recommended treatments, regardless of any patient’s preference or professional decision that led to the observed prescription choices. A detailed explanation of the methodology used for our cost simulation is presented in Appendix B.

## Results

### Trends in prescription and price

**Figure 1.**
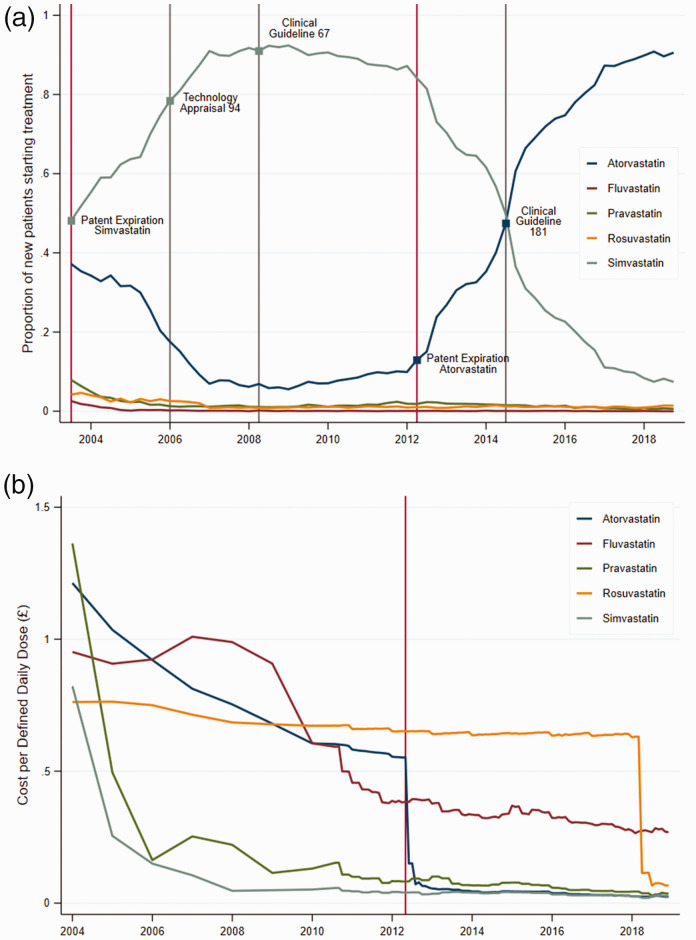
Trends in the statins prescribed for drug treatment initiation. (a) Proportion of new patients on each drug. (b) Average acquisition cost per defined daily dose (DDD) of each drug. Panel (a) shows the proportion of new patients starting drug treatment with each statin over time. The main five events are marked with vertical lines and small squares. The two vertical red lines marks the patent expiration of Zocor (simvastatin) and Lipitor (atorvastatin) in 2003 and May 2012, respectively; and the grey vertical lines indicate the publishing date of NICE’s statin-related national guidance. Panel (b) shows the average acquisition cost per DDD for each statin over time. Statins’ DDDs (or daily strength per day of treatment) established by the WHO are the following: for atorvastatin, 20 mg; fluvastatin 60 mg; pravastatin 30 mg; rosuvastatin 10 mg; and simvastatin 30 mg. Costs are obtained from Net Ingredient Cost figures from PCA, and are expressed in constant 2018 GBP using the GDP deflators at market prices, and money GDP from https://www.gov.uk/government/statistics/gdp-deflators-at-market-prices-and-money-gdp-march-2019-spring-statement. *Source*: Panel (a) from RCGP R&SC database, and panel (b) from Prescription Cost Analysis data series.

Figure 1(a) plots the evolution of the market shares for new patients starting treatment with statins between 2004 and 2018, using the RCGP R&SC database. In the time window considered, simvastatin and atorvastatin were the most frequently prescribed among the five statins, representing approximately 96% of all initial prescriptions. The dominance of these two drugs in treating cardiovascular diseases resulted in the evolution of their shares following mirror image patterns.

The share of simvastatin increased rapidly after its patent expiration in 2003, from around 50% to more than 90% in May 2008, when NICE published CG-67. While this guideline explicitly recommended simvastatin for treatment initiation, the percentage of new patients prescribed simvastatin stayed constant over the next four years up to May 2012, and, if anything, slightly decreased. We also note that the introduction of TA-94 in 2006 failed to accelerate the uptake of simvastatin. Upon atorvastatin’s patent protection expiration in May 2012, simvastatin’s share started decreasing steadily from around 85% in 2012 to around 10% in 2018. Once again, publication of CG-181 in 2014, updating the recommendation for treatment initiation to atorvastatin, had minimal effect in speeding up the declining trend of simvastatin.

Figure 1(b) shows the average acquisition costs per daily defined dose of each statin over time. The figure makes apparent the large drop in the acquisition cost of simvastatin soon after patent expiration of Zocor.^21^ Similarly, a sharp drop in acquisition cost for atorvastatin (virtually similar to simvastatin) was observed shortly after Lipitor patent expiration.

### Heterogeneity in prescriptions across general practices

**Figure 2.**
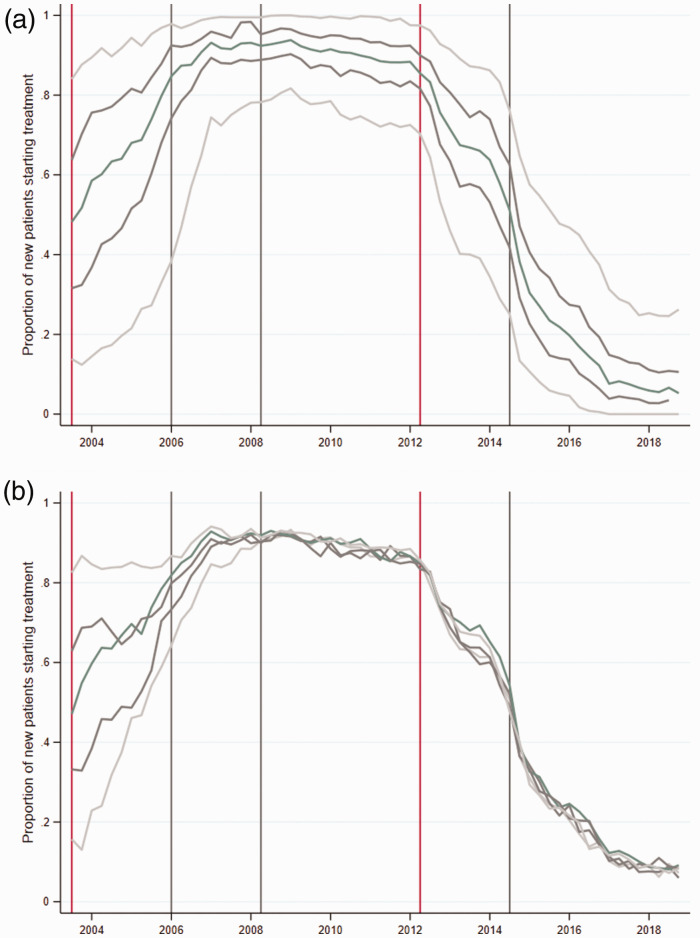
Heterogeneity in initial prescriptions at the general practice level. (a) Proportion of new patients on simvastatin with quantiles calculated quarterly. (b) Proportion of new patients on Simvastatin with fixed quantile composition calculated on Q3-2003. Panel (a) shows the average proportion of new patients treated with simvastatin within five quintiles of general practices ranked by proportion of simvastatin prescriptions (e.g. the top line represents the average proportion of patients initially treated with simvastatin, by the top 20th percent of general practices, etc.), where the quintiles of practices are obtained separately for each month (i.e. practices in each quantile may be different). Panel (b) shows the average proportion of new patients treated with simvastatin but for quintiles of practices obtained at Q3-2003, (i.e. the practices in each quintile are the same).* Source*: RCGP R&SC database.

Figure 2(a) presents average shares of new patients treated with simvastatin for each of the five quintiles of the general practices' prescription distribution. The figure reveals significant heterogeneity in prescribing choices across general practices during the time window of our study. When the simvastatin patent expired in May 2003, the proportion of patients treated with simvastatin ranged from less than 20% for general practices in the bottom quintile to more than 80% for the top quintile. The period up to 2006 saw an increase in the proportion of new patients treated with simvastatin across all general practices. At the time of the TA-94 introduction (January 2006), the difference between the second and fifth quintiles was around 20 percentage points, while the difference between first and fifth quintiles was still more than 50 percentage points. Following the introduction of CG-67 in May 2006, differences across practices fluctuated around 25 percentage points with most compliant with the NICE guideline practices treating almost all of their new patients with simvastatin, while least compliant practices prescribed simvastatin to less than 80% of their patients. Heterogeneity in prescription increased again following atorvastatin patent expiration in May 2012, when the difference in the share of new patients being prescribed simvastatin between the top and bottom quintiles reached about 50 percentage points. The subsequent CG-181 further reduced the overall levels of simvastatin prescriptions across the distribution but did little to reduce heterogeneity in the share of patients treated with simvastatin in the following years, with the difference between the top and bottom quintiles remaining at about 25 percentage points.

Figure 2(b) tracks the evolution of prescription for five quintiles of general practitioners as constructed in Q3-2003. The dynamics up to 2006 suggest the uptake of cost-effective prescribing for general practitioners in the lower quintiles is rather slow. However, the disappearance of major differences in prescribing among the five groups from 2007 onwards indicates that any given general practice does not systematically deviate from prescribing the cost-effective statin. These dynamics suggest that the overall heterogeneity observed in panel (a) is due to slow learning and fluctuation between cost-effective and non-cost-effective prescribing.

### Spending simulations

Spending simulation results for the ‘First Scenario’, which considered only the first prescription episode, are presented on the left-hand side panel of Table 1. Column (1) shows the estimated number of new patients taking statins in every year from Q3-2003 to Q4-2018. We observed a decrease in the number of new patients from 1.15 million in 2004 to 782,000 in 2018. The total cost of the first prescription episode for these new patients decreased from £26.9 million in 2004 to £862,000 in 2018 (a 96.8 percentage decrease), due to the reduction in the number of new patients as well as the acquisition costs of statins.

**Table 1.** Spending simulation exercise.

	First scenario					Second scenario				
	First prescription episode (first 28 days of treatment)					All prescriptions				
	(1)	(2)	(3)	(4)	(5)	(6)	(7)	(8)	(9)	(10)
Year	New patients (#)	Actual spending (£)	Hypothetical spending (£)	Savings (£)	Savings w.r.to actual spending (%)	All patients (#)	Actual spending (£m)	Hypothetical spending (£m)	Savings (£m)	Savings w.r.to actual spending (%)
2004	1,150,730	26,896,100	26,611,542	284,559	1.06	3,834,190	992.88	927.00	65.88	6.64
2005	1,024,085	13,164,249	10,332,805	2,831,444	21.51	4,457,033	758.85	586.94	171.91	22.65
2006	1,099,334	8,623,615	6,651,031	1,972,584	22.87	5,139,742	707.44	508.42	199.02	28.13
2007	905,206	4,771,157	3,128,028	1,643,129	34.44	5,548,000	627.31	288.83	338.48	53.96
2008	894,058	3,136,696	1,401,840	1,734,856	55.31	5,968,499	540.70	132.41	408.30	75.51
2009	873,406	3,254,101	1,415,472	1,838,629	56.50	6,351,952	527.68	126.14	401.54	76.10
2010	797,556	3,237,395	1,377,895	1,859,500	57.44	6,580,198	513.58	131.90	381.69	74.32
2011	733,701	3,145,095	1,029,339	2,115,756	67.27	6,670,907	494.57	104.61	389.95	78.85
2012	812,435	2,667,155	1,093,112	1,574,043	59.02	6,873,148	318.38	102.40	215.97	67.84
2013	804,797	1,416,539	1,402,155	14,384	1.02	7,103,662	164.28	132.90	31.38	19.10
2014	795,653	1,397,926	1,218,241	179,685	12.85	7,198,232	156.88	116.77	40.11	25.57
2015	792,381	1,486,687	1,310,250	176,437	11.87	7,316,021	163.59	124.42	39.16	23.94
2016	772,918	1,212,744	1,054,905	157,839	13.02	7,426,410	143.89	104.48	39.41	27.39
2017	801,680	1,150,362	940,154	210,208	18.27	7,526,470	138.76	92.57	46.19	33.29
2018	782,504	862,076	783,514	78,561	9.11	7,600,486	95.12	79.60	15.52	16.31
		76,421,897	59,750,283	16,671,614	21.82		6,343.91	3,559.39	2,784.51	43.89
										

Cost figures are expressed in constant 2018 GBP using the GDP deflators at market prices, and money GDP from https://www.gov.uk/government/statistics/gdp-deflators-at-market-prices-and-money-gdp-march-2019-spring-statement.

Overall, a saving of £16.67 million, or 21.8% of the total actual cost could have been realised for the first 28 days of treatment alone if general practitioners had prescribed simvastatin as initial drug treatment before May 2012 and atorvastatin after May 2012. Most of the hypothetical savings accrued over the period 2008–2012 when cheap generic versions of simvastatin became available and atorvastatin was still under patent protection. After May 2012, once generics of atorvastatin also entered the market, hypothetical savings on first prescription episodes were mainly accredited to switching from rosuvastatin to atorvastatin. However, the implied savings were not large as rosuvastatin only held a small share of the market.

Results for the ‘Second Scenario’ regarding total prescriptions for all existing patients are presented in the right-hand side panel of Table 1. The total number of patients on treatment every year increased from 3.8 million in 2004 to 7.6 million in 2018. However, the significant drop in price due to generic entries led to a drastic drop in spending over the same period: from just under £1 billion in 2004 to £95 million in 2018, a 90-percentage decrease. Cumulate spending on statins over the period 2004–2018 totalled £6.3 billion and estimated potential savings were £2.8 billion, or 43.9% of the actual spending on statins. As previously, large savings could have been obtained in the period 2004–2012 by switching patients from atorvastatin, which was available only as a branded drug, to simvastatin, that was generic.

## Discussion

In resource-constrained healthcare systems, promoting cost-effective prescribing behaviour is an important component of their cost-containment strategy. Using data on statins, we investigated how general practitioners' prescription choice in England changed in the face of (i) a large reduction in the cost of available treatments and (ii) the introduction of specific clinical guidelines. We demonstrated substantial increases in market shares for simvastatin and atorvastatin as their patents expired and generics entered the market, but well before the introduction of NICE guidelines recommending their use.

Those trends suggest that practitioners in primary care are sensitive to the price of alternative treatments, and that their choices even anticipate the recommendation of future clinical guidelines. Indeed, it took four years from the generic availability of simvastatin for NICE to explicitly recommend it for treatment initiation, by which point the share of new patients being prescribed the drug was already at 90%. Similarly, migration from simvastatin to atorvastatin started soon after a generic became available in May 2012, despite the fact that atorvastatin was only recommended as the preferred treatment in the updated NICE guideline two years afterwards.

Previous studies have shown that medicine management teams from CCGs do play a role in informing and influencing practices’ and practitioners’ prescription choices.^3–5^ Whether prescriptions are autonomously chosen by general practitioners or are influenced by the different actors within the primary care sector, our results suggest that ultimately prescribing decisions are more responsive to the acquisition cost of alternative treatments than clinical guidelines.

Although our analysis shows that, on average, practitioners treating patients at risk of cardiovascular events prescribed cost-effectively, we also identified substantial heterogeneity in prescription across practices, which remained even after the publication of official guidelines. Our descriptive analysis indicates some general practitioners took longer to adopt cost-effective prescribing and some switched in and out of cost-effective prescribing throughout the study period, generating large overall heterogeneity. A number of explanations can be offered for such behaviours. For example, medicolegally, general practitioners may have felt inhibited to change prescribing habits simply on the basis of cost, without having had guidelines to justify the decision.^22^ Equally, general practitioners' and patients’ characteristics, practices’ characteristics, geo-social conditions, as well as local CCG prescribing guidelines and monitoring activities may influence prescribing decisions.^23^ Moreover, statins have been widely perceived as causing side effects such as muscle pains (with intermittent media coverage influencing prescribing behaviour).^24^ General practitioners and patients may have been reluctant to switch statins for fear of inducing adverse events.^25^

Under the plausible assumption that general practitioners cannot consistently anticipate whether a *new* patient would benefit from taking a drug other than the one recommended by NICE, we evaluated prescription choices in this market according to a cost-minimisation criterion where choosing statins other than simvastatin (before May 2012) or atorvastatin (after May 2012) can be considered suboptimal. Our cost-savings simulation analysis suggested that low responsiveness comes at a high price for the NHS. Namely, if all new patients had received the most cost-effective treatment (as later recommended in the guidelines), the NHS could have saved around 22% of the actual spending on initial prescriptions. Looking at all prescriptions for new and on going patients, we compute savings of £2.7bn, mainly between 2004 and 2012, representing roughly 44% of total spending on statins during this period. We acknowledge that this figure is an upper bound of potential savings, based on the strong assumption that all existing patients on drug treatment could be seamlessly switched to other statins, without considering side effects (e.g. myopathy) or other practicalities (e.g. planned-patient reviews) . Looking at the data, we found that around 7% and 12% of patients were switched to simvastatin and then switched away from it in the next 4 and 12 months, respectively. Although these numbers suggest that simvastatin cannot be used by a non-insignificant proportion of patients, there is no doubt that there were still large savings to be made by prescribing the most cost-effective statins.

We anticipate that the experience of statins would be similarly observed in other therapeutic areas where treatments have similar modes of action and comparable levels of efficacy, for instance angiotensin-converting enzyme (ACE) inhibitors and proton pump inhibitors. Looking ahead, our analysis suggests that cost-conscious centralised public health systems could save substantial sums if new and, whenever possible, on going patients are promptly switched to cost-effective alternatives, in particular when the first medicine in a therapeutic class loses patent protection. The observed heterogeneity in prescribing behaviour suggests that an important step forward towards achieving this goal would be a timely dissemination of best practices, with the aim of promoting cost-conscious prescribing behaviour. In the UK, where general practices are grouping into Primary Care Networks and there is growing co-working and co-location with pharmacists, such collaborative efforts are likely to drive future prescribing. Given general practitioners' limited time available to acquire information on market developments (e.g. new medicines coming into the market or brand-name medicines losing patent protection) across all drug classes they prescribe, there is an important role for academic detailing as well as online/computerised systems and prompts such as ScriptSwitch, rather than paper-based (e.g. Prescribing Outlook), to educate and offer updated advice on cost-effective medicines while preserving physicians’ freedoms to prescribe and patients’ ability to discuss their preferred choice of treatment.^26^ It is of note that the recently announced NICE strategy for 2021 to 2026 aims to ‘provide dynamic, living guideline recommendations that are useful, useable and rapidly updated’ (p. 19).^27^

### Strengths and limitations

We study prescription dynamics of statins, a class of drugs widely prescribed in primary care, using a representative dataset of English practices for the period 2004–2018, a time window that includes patent expiration of brand-name statins and publications of new NICE guidelines. There is no reason to believe that the large forsaken savings we have identified would not generalise to other important therapeutic areas of the English NHS or to other healthcare systems. Admittedly, the extent of the savings is an empirical matter and crucially depends on the structure of healthcare systems, the penetration of generics within them and the incentives of different players in prescribing, dispensing, and reimbursing pharmaceutical treatments.

We acknowledge a few limitations for this study. We only observe a first prescription issued to patients treated in primary care, without being able to account for prescribing influences coming from other settings. For example, patients experiencing a first cardiovascular event may have received their first statin prescription in secondary care, and such decision might have influenced ongoing prescribing in primary care. However, it is unlikely that this would explain all of the heterogeneity in prescribing choices and the large forsaken savings shown in Table 1. Further, we had access only to limited data on general practitioner characteristics to examine factors associated with the observed heterogeneity, while analysis of free text from clinical records to explore documented decisions related to statin prescription choice was beyond the scope of this project.

## Conclusions

The fact that general practitioners react to prices illustrates the strengths of a healthcare system that pays attention to cost-effectiveness. There is potential for large savings for the NHS if new and, whenever possible, on going patients are promptly switched to the first medicine that becomes available as generic within a therapeutic class where all other medicines have similar efficacy. On going efforts to create a system infrastructure to support and monitor general practitioner prescribing locally could prove effective in aligning incentives to select cost-effective treatments while preserving physicians’ freedoms to prescribe and patients’ ability to discuss their preferred choice of treatment. [Table table2-01410768211051713][Table table3-01410768211051713]

**Table B1. table2-01410768211051713:** Percentage reduction in low-density lipoprotein cholesterol.

Statin	Dose mg/day				
	5	10	20	40	80
Fluvastatin			21%	27%	33%
Pravastatin		20%	24%	29%	
Simvastatin		27%	32%	37%	42%
Atorvastatin		37%	43%	49%	55%
Rosuvastatin	38%	43%	48%	53%	

20%–30%: low intensity; 31%-40%: medium intensity; above 40%: high intensity.

**Table B2. table3-01410768211051713:** Correspondence between all statins’ treatments based on low-density lipoprotein cholesterol reduction.

(1)	(2)	(3)	(4)
Original treatments		Similar treatments	
Statin	mg/day	Simvastatin	Atorvastatin
Atorvastatin	10	40	
	20	80	
	30	80	
	40	80	
	60	80	
	80	80	
Fluvastatin	20	10	10
	40	10	10
	80	20	10
Pravastatin	10	10	10
	20	10	10
	40	10	10
Rosuvastatin	5	40	10
	10	80	20
	20	80	40
	40	80	80
Simvastatin	10		10
	20		10
	40		10
	80		20

This table is based on the Grouping from Table B1.

## Supplemental Material

sj-pdf-1-jrs-10.1177_01410768211051713 - Supplemental material for Price versus clinical guidelines in primary care statin prescribing: a retrospective cohort study and cost simulation model Click here for additional data file. Supplemental material, sj-pdf-1-jrs-10.1177_01410768211051713 for Price versus clinical guidelines in primary care statin prescribing: a retrospective cohort study and cost simulation model by Matias Ortiz De Zarate, Emmanouil Mentzakis, Simon DS Fraser, Paul Roderick, Paul Rutter and Carmine Ornaghi in Journal of the Royal Society of Medicine

## Figures and Tables

**Figure A1.**
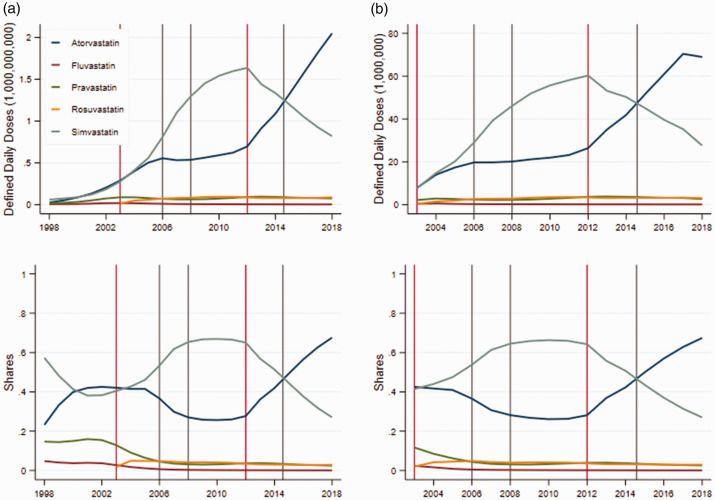
Volume of statins prescribed in main data sources. (a) Prescription Cost Analysis data. (b) RCGP R&SC data.

## Declarations

**Ethical approval:** The study was approved by the Ethics Committee of the University of Southampton (submission ID 52995). The nature of the research question does not pose threats to the privacy rights of patients and general practices.

**Guarantor:** Carmine Ornaghi.

**Contributorship:** MO, EM and CO contributed to the design of the study. MO extracted the data and wrote the statistical programmes. CO, EM and MO wrote the first draft. All authors contributed to further drafts, conceptualisation and approved the final manuscript. CO is guarantor for the work.

**Data sharing:** The data were obtained from the Royal College of General Practitioners Research and Surveillance Centre (RCGP R&SC). Data were extracted in January 2019 and analysis was performed from a laboratory at the University of Surrey through a secure network and remote desktop connection. RCGP R&SC data governance and our own license to use those data do not allow us to distribute or make available patient data directly to other parties. Researchers can apply for data access at https://www.rcgp.org.uk/clinical-and-research/our-programmes/research-and-surveillance-centre/supporting-research-teams/submit-a-data-request-online-form.aspx.

**Transparency:** The corresponding author affirms that the manuscript is an honest, accurate and transparent account of the study being reported, and that no important aspects of the study have been omitted.

## Appendix

### Appendix A

In this Appendix, we compare the data in the Prescription Cost Analysis (PCA) dataset to those in the RCGP R&SC dataset to demonstrate that the latter constitutes a representative sample of national prescription. One advantage of the PCA database is that prescription data go back to the year 1998. However, the PCA database cannot be used to investigate heterogeneity in prescription choice because data are available only at national level, not at general practice level.

Figure A1 compares the data over time in our two data sources: Panel (a) on the left shows the figures from the PCA dataset between 1998 and 2018. Panel (b) on the right shows the figures from RCGP R&SC database from 2004 to 2018. Top panels display the total quantity in terms of daily defined doses (DDDs) while the bottom panels display the shares of each of the five statins in the market. The similarity in the trends reported in panel (a) and (b) confirms that the RCGP R&SC database is a representative sample of national data of statins prescription.

### Appendix B

**Spending savings simulation exercise methodology:** This appendix describes the methodology used for the spending savings simulation exercise, by which we estimate the potential savings for the NHS that could have been achieved if general practitioners had prescribed simvastatin or atorvastatin as active ingredients, whenever these two medicines were the prescribing standard in this market according to the observed preferences of general practitioners and the recommendations in national guidance. We start by describing the computations of actual and hypothetical cost for the *first scenario*, in which the analysis refers to the first prescription episode, i.e. the first 28 days of drug treatment, for *new* patients only; and then the *second scenario*, in which we apply the same methodology to all prescriptions issued to *all* existing patients being treated in every period.

### First scenario

To compute actual and hypothetical cost, we use information on the number of new patients treated, their initial drug treatment (i.e. a specific statin and strength), and a measure of each treatment’s acquisition cost to the NHS per day of treatment. From the RCGP R&SC database, we count the number of new patients being prescribed statin treatment *s* in period *t* for the first time, denoted by $n_{\mathrm{st}}$ .

From the Prescription Cost Analysis (PCA) series, containing data on all medicines prescribed and dispensed and their corresponding cost to the NHS at the national level, we retrieve a measure of the actual acquisition cost of each statin treatment. To compute the average acquisition cost of treatment *s* in period *t*, denoted by $C_{\mathrm{st}}$ , we take the ratio between the Net Ingredient Cost ( ${\mathrm{NIC}}_{\mathrm{st}}$ ) and the corresponding Total Quantity ( $Q_{\mathrm{st}}$ ) prescribed of each different strength of statin, that is $C_{\mathrm{st}}=\frac{{\mathrm{NIC}}_{\mathrm{st}}}{Q_{\mathrm{st}}}$ . Cost figures are then expressed in constant 2018 GBP using the GDP deflators at market prices (see https://www.gov.uk/government/statistics/gdp-deflators-at-market-prices-and-money-gdp-march-2019-spring-statement).

Since data on the total number of new patients starting treatment on each statin nationally are not publicly available, we estimate such figure by combining information from the RCGP R&SC database (which is a nationally representative sample of general practices in England) with national aggregated data from the Prescription Cost Analysis series. Concretely, we compute the total number of new patients nationally, denoted by $\overset{\wedge }{N}$ , as follows: ${\overset{\wedge }{N}}_{\mathrm{st}}=\frac{n_{\mathrm{st}}}{q_{\mathrm{st}}}\times Q_{\mathrm{st}}$ , where $q_{\mathrm{st}}$ denotes the total quantity of each statin treatment prescribed in every period in the RCGP R&SC database. Indeed, since the RCGP R&SC sample of GP practices is representative of the English general practice sector, then the ratio of new patients to total quantities prescribed in both data sources should be equivalent. Aggregating ${\overset{\wedge }{N}}_{\mathrm{st}}$ over all treatments at the year-level, ${\overset{\wedge }{N}}_{t}=\sum_{s}{\overset{\wedge }{N}}_{\mathrm{st}}$ , results in the figure reported in column (1) of Table 1, i.e. the estimated number of new patients treated with statins in each year.

Finally, we compute the actual cost of first prescription episodes for each statin treatment *s* in every period *t* by multiplying the total number of new patients on each treatment ${\overset{\wedge }{N}}_{\mathrm{st}}$ with the cost per day of treatment $C_{\mathrm{st}}$ times 28, that is ${\mathrm{AC}}_{\mathrm{st}}={\overset{\wedge }{N}}_{\mathrm{st}}\times C_{\mathrm{st}}\times 28$ . Then we aggregate ${\mathrm{AC}}_{\mathrm{st}}$ over all treatments at the year-level, ${\mathrm{AC}}_{t}=\sum_{s}{\mathrm{AC}}_{\mathrm{st}}$ , which is the figure reported in column (2) of Table 2.

As explained above, practitioners’ preferences when treating patients for cardiovascular disease risk moved towards simvastatin from its patent expiration (May 2003) until atorvastatin’s patent expiration (May 2012); and from then onwards, they tended towards atorvastatin. Our cost simulation exercise *extremes* this observed behaviour by asking what would have been the cost savings if either simvastatin or atorvastatin had been the active ingredients originally prescribed to new patients, whenever these two medicines were the prescribing standard in specific periods. Accordingly, the hypothetical cost is constructed by substituting the originally prescribed treatment *s*, with a *therapeutically similar* one, denoted by $s^{*}$ , containing simvastatin for those first-time prescriptions issued between 2004 and May 2012, or atorvastatin for those issued after May 2012.

The therapeutic similarity criteria we use is based on the ability of each strength of each drug (e.g. 1 tablet of atorvastatin 20 mg a day, 1 tablet of simvastatin 40 mg a day, etc.) in reducing low-density lipoprotein cholesterol levels per day of treatment. The percentage reduction in low density lipoprotein cholesterol is used in NICE’s CG-181 to group the five statins (and each of their corresponding strengths) according to their intensity. The relationship between the strengths of the statins and reduction in low-density lipoprotein cholesterol is stated in NICE's CG-181, which in turn is based on the paper by Law et al.^16^ A reproduction of this information is presented in Table B1.

To make this operative, for each level of percentage reduction in low-density lipoprotein cholesterol achieved by the originally prescribed treatment, i.e. a drug-strength pair, we look for the closest strength of both simvastatin and atorvastatin that achieves a similar level in low-density lipoprotein reduction to the originally prescribed one. The correspondence between original treatments and the substitutes is presented in Table B2. Columns (3) and (4) show the strength of simvastatin and atorvastatin, respectively, that achieves the closest percentage reduction in low-density lipoprotein cholesterol than the original treatments listed in columns (1) and (2). For example, if a patient was prescribed atorvastatin 10 mg a day for treatment initiation before 2012, the hypothetical prescription for this patient is a treatment of simvastatin 40 mg a day, as both achieve a reduction of 37% in low-density lipoprotein cholesterol. Second example, if a patient was prescribed rosuvastatin 10 mg for treatment initiation after 2012, then the hypothetical prescription for this patient is a treatment of atorvastatin 20 mg, as both achieve a reduction of 43%.

Finally, the hypothetical cost is computed by multiplying the total number of new patients times the cost of the therapeutically similar treatments $C_{s^{*}t}$ times 28, that is ${\mathrm{HC}}_{\mathrm{st}}={\overset{\wedge }{N}}_{\mathrm{st}}\times C_{s^{*}t}\times 28$ . Then we aggregate ${\mathrm{HC}}_{\mathrm{st}}$ over all treatments at the year-level, ${\mathrm{HC}}_{t}=\sum_{s}{\mathrm{HC}}_{\mathrm{st}}$ , which is the figure reported in column (3) of Table 2.

**Second scenario:** The second scenario considers not only first-time prescriptions for new patients, but all prescriptions for all existing patients treated with statins. For this, we use the information on total quantity and spending from the PCA database. The actual cost is obtained by aggregating spending on all statins prescribed in each year. The hypothetical cost is computed by replacing the per unit cost of the original treatment (statins and strength) with the corresponding cost of the therapeutically similar treatment (either simvastatin or atorvastatin), as described above. Additionally, to provide an estimate of the total number of all existing patients treated with statins in every period, ${\overset{\wedge }{P}}_{t}$ (the figure reported in column (6) of Table 1), we compute ${\overset{\wedge }{P}}_{t}=\sum_{s}\frac{p_{\mathrm{st}}}{q_{\mathrm{st}}}\times Q_{\mathrm{st}}$ , where $p_{\mathrm{st}}$ denotes the total number of all existing patients using treatment *s* at time *t* as reported in the RCGP R&SC database.
