# Supplementary material for: Price versus clinical guidelines in primary care statin prescribing: a retrospective cohort study and cost simulation model
Source: J R Soc Med. 2021 Nov 18;115(3):100–11. doi: 10.1177/01410768211051713 (PMC8981530; doi:10.1177/01410768211051713)
Supplement: sj-pdf-1-jrs-10.1177_01410768211051713 - Supplemental material for Price versus clinical guidelines in primary care statin prescribing: a retrospective cohort study and cost simulation model [file sj-pdf-1-jrs-10.1177_01410768211051713.pdf]

APPENDIX A

In this Appendix we compare the data in the PCA dataset to those in the RCGP R&SC dataset to demonstrate that the latter constitutes a representative sample of national prescription. One advantage of the PCA database is that prescription data go back to the year 1998. However, the PCA database cannot be used to investigate heterogeneity in prescription choice because data are available only at national level, no at GP practice level.

Figure A1 compares the data over time in our two data sources: Panel (a) on the left shows the figures from the PCA dataset between 1998-2018. Panel (b) on the right shows the figures from RCGP R&SC database from 2004-2018. Top panels display the total quantity in terms of daily-defined-doses (DDDs) while the bottom panels display the shares of each of the five statins in the market. The similarity in the trends reported in panel (a) and (b) confirms that the RCGP R&SC database is a representative sample of national data of statins prescription.

Figure A1: Volume of statin's prescribed in main data sources

(a) Prescription Cost Analysis data (b) RCGP R&SC data

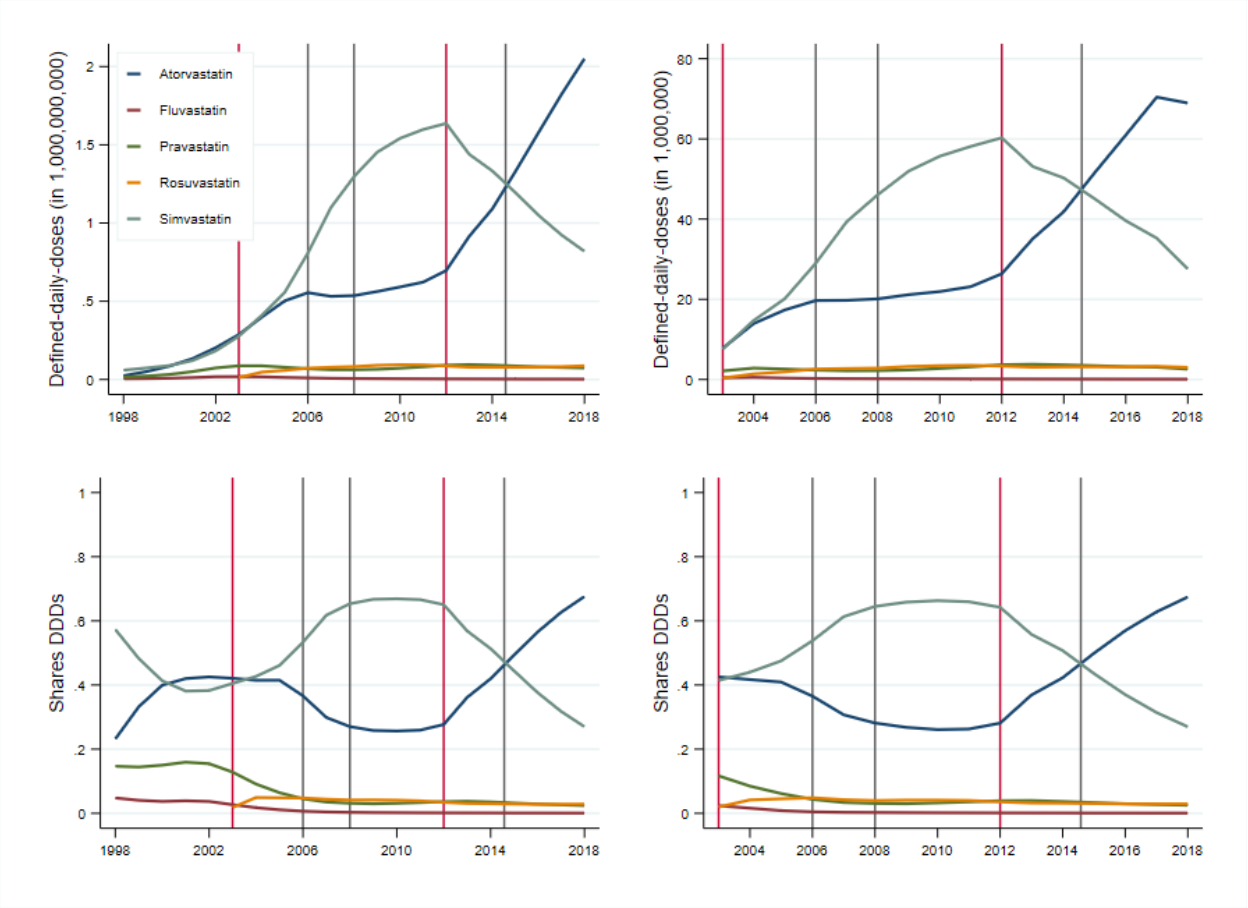

APPENDIX B

Spending Savings Simulation Exercise Methodology

This appendix describes the methodology used for the spending savings simulation exercise, by which we estimate the potential savings for the NHS that could have been achieved if GPs had prescribed simvastatin or atorvastatin as active ingredients, whenever these two medicines were the prescribing standard in this market according to the observed preferences of GPs and the recommendations in national guidance. We start by describing the computations of actual and hypothetical cost for the *first scenario*, in which the analysis refers to the first prescription episode, i.e., the first 28 days of drug treatment, for *new* patients only; and then the *second scenario*, in which we apply the same methodology to all prescriptions issued to *all* existing patients being treated in every period.

First scenario

To compute actual and hypothetical cost we use information on the number of new patients treated, their initial drug treatment (i.e., a specific statin and strength), and a measure of each treatment's acquisition cost to the NHS per day of treatment. From the RCGP R&SC database, we count the number of new patients being prescribed statin treatment *s* in period *t* for the first time, denoted by n_{st}.

From the Prescription Cost Analysis (PCA) series, containing data on all medicines prescribed and dispensed and their corresponding cost to the NHS at the national level, we retrieve a measure of the actual acquisition cost of each statin treatment. To compute the average acquisition cost of treatment *s* in period t, denoted by C_{st}, we take the ratio between the Net Ingredient Cost (NIC_{st}) and the corresponding Total Quantity (Q_{st}) prescribed of each different strength of statin, that is $C_{st} = \frac{NIC_{st}}{Q_{st}}$. Cost figures are then expressed in constant 2018 GBP using the GDP deflators at market prices (see https://www.gov.uk/government/statistics/gdp-deflators-at-market-prices-and-money-gdp-march-2019-spring-statement)

Since data on the total number of new patients starting treatment on each statin nationally is not publicly available, we estimate such figure by combining information from the RCGP R&SC database (which is a nationally representative sample of GP practices in England) with national aggregated data from the Prescription Cost Analysis series. Concretely, we compute the total number of new patients nationally, denoted by $\widehat{N}$, as follows: $\widehat{N}_{st} = \frac{n_{st}}{q_{st}} \times Q_{st}$, where q_{st} denotes the total quantity of each statin treatment prescribed in every period in the RCGP R&SC database. Indeed, since the RCGP R&SC sample of GP practices is representative of the English general practice sector, then the ratio of new patients to total quantities prescribed in both data sources should be equivalent. Aggregating $\widehat{N}_{st}$ over all treatments at the year-level, $\widehat{N}_t = \sum_s \widehat{N}_{st}$, results in the figure reported in column (1) of Table 1, i.e., the estimated number of new patients treated with statins in each year.

Finally, we compute the actual cost of first prescription episodes for each statin treatment *s* in every period t by multiplying the total number of new patients on each treatment $\widehat{N}_{st}$ with the cost per day of treatment C_{st} times 28, that is $AC_{st} = \widehat{N}_{st} \times C_{st} \times 28$. Then we aggregate AC_{st} over all treatments at the year-level, $AC_t = \sum_s AC_{st}$, which is the figure reported in column (2) of Table 1.

As explained above, practitioners' preferences when treating patients for CVDs risk moved towards simvastatin since its patent expiration (May 2003) until atorvastatin's patent expiration (May 2012); and from then onwards, they tended towards atorvastatin. Our cost simulation exercise *extremes* this observed behaviour by asking what would have been the cost savings if either simvastatin or atorvastatin

had been the active ingredients originally prescribed to new patients, whenever these two medicines were the prescribing standard in specific periods. Accordingly, the hypothetical cost is constructed by substituting the originally prescribed treatment s, with a *therapeutically similar* one, denoted by s^*, containing simvastatin for those first-time prescriptions issued between 2004 and May 2012, or atorvastatin for those issued after May 2012.

The therapeutic similarity criteria we use is based on the ability of each strength of each drug (e.g., 1 tablet of atorvastatin 20 mg. a day, 1 tablet of simvastatin 40 mg. a day, etc.) in reducing low-density lipoprotein (LDLP) cholesterol levels per day of treatment. The percentage reduction in low density lipoprotein cholesterol is used in NICE’s CG-181 to group the five statins (and each of their corresponding strengths) according to their intensity. The relationship between the strengths of the statins and reduction in LDLP cholesterol is stated in NICE's CG-181, which in turn is based on the paper by Law et al. (2003).[16] A reproduction of this information is presented in Table B1.

Table B1: Percentage Reduction in low-density lipoprotein cholesterol

Statin	Dose [mg.] per day				
	5	10	20	40	80
Fluvastatin			21%	27%	33%
Pravastatin		20%	24%	29%	
Simvastatin		27%	32%	37%	42%
Atorvastatin		37%	43%	49%	55%
Rosuvastatin	38%	43%	48%	53%	

Notes. 20%-30%: low intensity. 31%-40%: medium intensity. Above 40%: high intensity.

To make this operative, for each level of percentage reduction in LDLP cholesterol achieved by the originally prescribed treatment, i.e., a drug-strength pair, we look for the closest strength of both simvastatin and atorvastatin that achieves a similar level in LDLP reduction to the originally prescribed one. The correspondence between original treatments and the substitutes is presented in Table B2. Columns (3) and (4) show the strength of simvastatin and atorvastatin, respectively, that achieves the closest percentage reduction in LDLP cholesterol than the original treatments listed in columns (1) and (2). For example, if a patient was prescribed atorvastatin 10 mg a day for treatment initiation before 2012, the hypothetical prescription for this patient is a treatment of simvastatin 40 mg a day, as both achieve a reduction of 37% in LDLP cholesterol. Second example, if a patient was prescribed rosuvastatin 10 mg for treatment initiation after 2012, then the hypothetical prescription for this patient is a treatment of atorvastatin 20 mg, as both achieve a reduction of 43%.

Table B2: Correspondence between all Statins' treatments based on LDL cholesterol reduction

(1)	(2)	(3)	(4)
Original Treatments		Similar Treatments	
Statin	Strength [mg.]	Simvastatin	Atorvastatin
Atorvastatin	10	40	
	20	80	
	30	80	
	40	80	
	60	80	
	80	80	
Fluvastatin	20	10	10
	40	10	10
	80	20	10
Pravastatin	10	10	10
	20	10	10
	40	10	10
Rosuvastatin	5	40	10
	10	80	20
	20	80	40
	40	80	80
Simvastatin	10		10
	20		10
	40		10
	80		20

Notes. This table is based on the Grouping from Table B1.

Finally, the hypothetical cost is computed by multiplying the total number of new patients times the cost of the therapeutically similar treatments C_{s^*t} times 28, that is $HC_{st} = \widehat{N}_{st} \times C_{s^*t} \times 28$. Then we aggregate HC_{st} over all treatments at the year-level, $HC_t = \sum_s HC_{st}$, which is the figure reported in column (3) of Table 1.

Second scenario

The second scenario considers not only first-time prescriptions for new patients, but all prescriptions for all existing patients treated with statins. For this, we use the information on total quantity and spending from the PCA database. The actual cost is obtained by aggregating spending on all statins prescribed in each year. The hypothetical cost is computed by replacing the per unit cost of the original treatment (statins and strength) with the corresponding cost of the therapeutically similar treatment (either simvastatin or atorvastatin), as described above. Additionally, to provide an estimate of the total number of all existing patients treated with statins in every period, $\widehat{P}_t$ (the figure reported in column (6) of Table 1), we compute $\widehat{P}_t = \sum_s \frac{p_{st}}{q_{st}} \times Q_{st}$, where p_{st} denotes the total number of all existing patients using treatment *s* at time *t* as reported in the RCGP R&SC database.
